# Evaluation of apap parameters in one to three nights titration for final pressure decision-making in a real world clinical setting

**DOI:** 10.1007/s11325-025-03481-2

**Published:** 2026-05-07

**Authors:** Jesús Escribá-Alepuz, Lidia Herrero, Alberto Saura, Amparo Lloris, Oldar Cercas, José Segrelles, Jorge Pinel, Marta Palop, Gemma Ramos, Estefania Estévez, F. Javier Puertas

**Affiliations:** 1 Neurophysiology Unit, Sagunto Hospital, Valencia, Spain; 2 Sleep Medicine Institute, Valencia, Spain; 3 Department of Health Psychology, Miguel Hernandez University (MHU), Elche, Spain; 4 National Center for Epidemiology, Carlos III Health Institute, Madrid, Spain; 5 Pneumology Unit, Sagunto Hospital, Valencia, Spain; 6 Air Liquide, Valencia, Spain; 7 Sleep Unit, La Ribera University Hospital - FISABIO, Valencia, Spain; 8 Faculty of Medicine, Catholic University of Valencia, Valencia, Spain

**Keywords:** OSA, Auto-titration, APAP, CPAP

## Abstract

**Purpose:**

Compare pressure values obtained from APAP titration with the final pressure (PF) selected by the clinician to approximate and optimize as much as possible our OSA patients treatment management.

**Methods:**

Retrospective descriptive study in 114 OSA treated patients. After 2–3 months with empiric CPAP pressure, they underwent 2–3 nights with 2 different APAP devices to help clinicians to decide an APAP pressure according to percentile 95 (P95) and median (Pm) values after visual analysis. We selected 60 patients (30 for each device) meeting the inclusion criteria until our 3 months follow-up consultation when, according to clinical outcomes, final pressure (PF) is established. Data analysis was blind and random, by an experienced and independent sleep physician and how and why the clinicians used the APAP was statistically evaluated, measuring parameters from each device for every patient and combination of nights and comparing them with PF, whose correlation with AHI was also analyzed.

**Results:**

We found only significant evidence (*p* < 0.05) using Pm and Resmed device and considering AHI as a compliance predictive value, but no statistical difference (*p* > 0.05) performing more than one APAP night.

**Conclusions:**

A single night of APAP titration and use of the median pressure may suffice to determine an optimal fixed CPAP setting in our patients. We discuss limitations including small sample size, exclusion of unstable patients and absence of laboratory PSG comparison and Spanish-specific practice patterns.

## Introduction

Obstructive sleep apnea (OSA) is a common chronic disorder with significant health consequences, affecting up to ~ 1 billion people worldwide [1]. The standard treatment for moderate-to-severe OSA is continuous positive airway pressure (CPAP) during sleep, which consistently reduces apneas, daytime symptoms, and cardiovascular risk factors [1, 2]. However, determining the optimal fixed CPAP pressure traditionally requires an in-laboratory titration polysomnography as a gold-standard procedure, which is resource-intensive and often limited by availability [1–3, 3], because it requires the continuous presence of a sleep technician, usually in a specialized sleep unit, what justifies the need of other validated methodologies, to solve this frequent and significant public health problem [4].

As a result, many centers use home-based strategies: portable sleep studies for diagnosis and auto-titrating CPAP (APAP) devices for pressure selection to avoid long waiting lists, frequent repetition of studies and find better cost-effective procedures [5, 6] and compliance [7]. In fact, recently, there have been several attempts to simplify this issue by using different devices [8, 9] formulas [10] and other methods [11, 12], even testing other another sleep tests [13].

The concept of auto-titration continuous positive airway pressure (APAP) has become the routine approach to OSA long-term treatment in many countries, as it should improve its efficacy and compliance by resolving the problems of intra and inter-night variability of pressure requirements [9], although there are many other possible factors whose influence is yet widely to be confirmed [5, 8, 14, 15].

On the other hand, self-piloted PAP would also eventually eliminate the need for PSG or optimal fixed-titration. However, their efficacy and long-term superiority over continuous pressure devices is an unresolved issue [3, 16, 17].

Several clinical trials have been performed with APAP in different modalities in attended or unattended conditions, or as a definitive treatment device itself, also considering the better comfort and adherence that they provide to the patient, but literature concerning this topic is still limited [18–21].

Comparison of these trials is challenging, since APAP technology is evolving promptly and devices differ in how their operational algorithm responds accordingly, whose details are patent protected and sometimes difficult to understand and modify by the clinicians.

Auto-titration has been proposed as a common standard practice in OSA titration [17, 21, 22] and treatment [6, 19, 21, 23] because no significant differences on residual AHI, compliance and use were found and an improvement in the Epworth Sleepiness Scale (ESS) or the Functional Outcomes of Sleep Questionnaire (FOSQ) for APAP treatment has been described with regard to CPAP treatment in some samples [22, 23]. In fact, especially in response to COVID-19 and resource constraints, home APAP titration has been more globally adopted [19] and, although each APAP manufacturer uses proprietary algorithms and event definitions, APAP usually tends to use slightly lower mean pressure, deliver higher peak pressures and APAP-reported pressures and indices may not align precisely with polysomnography-based metrics. Because of these differences, professional guidance suggests interpreting APAP-derived values (like 95th percentile pressure or median pressure) with caution when setting a fixed pressure.

Unfortunately, there are not already many studies evaluating different APAP machines with other titration methods [10, 24–27], optimal number of titration nights, clinical approach and decision making process of pressure selection in a real world setting.

In Spain, approximately 5–7 million people have OSA, with ~ 2 million requiring CPAP therapy what is delivered through a public health system without copayment from patient’s side, this implies that the routine PAP machines that patients receive are CPAP. AutoCPAP are still limited as routine long-term treatment for special cases of high pressures or poor CPAP tolerance, also cost-free for patients. However, the use of AutoCPAP devices as a titration method for obtaining the fixed pressure is very common in a home basis, with a 2–3 nights test and a later reviewing of software data by the clinician [28]. So Spain’s approach emphasizes ambulatory diagnosis and treatment for high pre-test probability non co-morbid OSA patients, multi-night home trials and specialist consultation review and follow up, but this context differs from other healthcare systems.

##  Objectives

The main objective was the comparison between the most important APAP titration parameters (P95 and PMed) obtained from two different devices and the definitive CPAP value selected by the clinician (PF), after an adaptation period with an empiric CPAP pressure (Pe) and a complete follow-up in compliant OSA patients following our standard clinical, diagnostic and therapeutic approach.

As secondary purposes, we intended to study how many titration nights are necessary to assure an optimal approximation to the PF.

Furthermore, we analyzed the differences between two different APAP devices and assessed the value of the home sleep test AHI’s as a compliance predictive factor.

## Methods

We performed a retrospective descriptive study in 114 consecutive OSA patients diagnosed by home ambulatory polygraphy as a home-sleep testing (HST, level III AASM), referred between June 2018 and December 2019 because of snoring and/or excessive daytime sleepiness (Epworth Scale > 12) and treated afterwards with CPAP, attended in the sleep consultation of the Respiratory Department in Sagunto University Hospital, Valencia (Spain).

All patients had an CPAP trial of 2–3 months duration with and empiric pressure (Pe, determined by Miljeteig and Hoffstein formula [12]). After this adaptation period, each patient underwent random home APAP titration using one of two available APAP devices in our hospital (included in our public health for free: device 1 or Breas iSleep i20i and device 2 or Resmed Autoset S7) over 2 or 3 consecutive nights. In practice, Breas device was used for 26 patients with 3-night studies and 4 patients with 2-night studies; Resmed device was used for 18 patients with 3 nights and 12 patients with 2 nights.

**Fig. 1:**
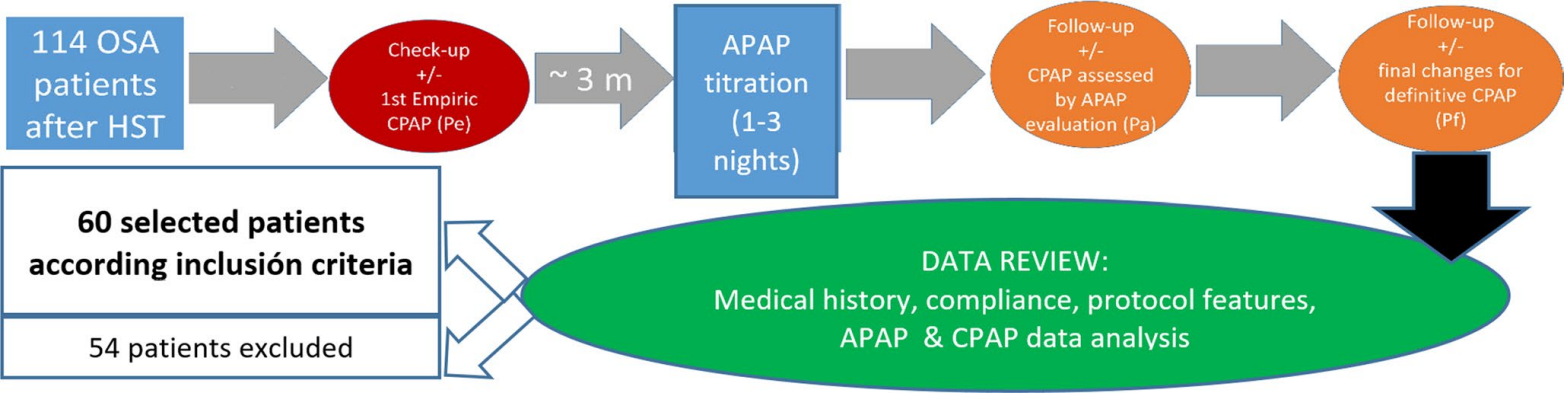
Diagram 1. Simplified illustration of the protocol for participants

These devices recorded detailed pressure data overnight and we extracted for each night of APAP titration, the 95th percentile pressure or P95 (pressure levels that eliminate breathing obstructions for at least 95% of the sleep period) and the median pressure or Pmed (arithmetic median of all pressure values) from the APAP software both from each patient and night, in order to guide our clinicians about which is the best APAP titration strategy according our protocol (fig. 1) in a follow-up consultation, when a fixed pressure (Pa) is established taking in account all these APAP parameters and patient adaptation and response to previous empiric pressure (Pe).

Then, after 3 months, there was a last consultation when the final pressure (PF) was determined, according to clinical outcomes and protocols used in other similar studies [10, 29], pressure could be decreased to improve their compliance or increased by 1 or 2 cmH2O if the patient’s partner observed remaining snoring.

Retrospectively and after more than 1 year after this final pressure (PF) was prescribed, medical appointments and history, previous CPAP compliance and APAP titration data were studied with a blind, random and manual accurate review by an independent experienced sleep expert external to the Respiratory Department for 114 patient’s records who meet these inclusion criteria:

- Not previously treated with APAP.
- Compliants with the treatment (use of CPAP > 4 h/day for at least 70% of the nights during 3 consecutive months) and the clinical protocol (tests and consultations performed without delays and assistance problems).
- Evaluated with a minimum of 2 valid APAP night without periods in which the total leak didn’t’ exceed 25 L/min or 0.4 L/s and multiple pressure variations, and taking into account a possible residual AHI less than 5 events per hour.
- 1 year treated with a stable optimal final pressure (PF) and without health care needed.

Finally, 60 patients were selected (Table 1) according the defined inclusion criteria and 54 patients were excluded (Table 2), because of:

- Adaptation problems or poor CPAP compliance (< 4 h)
- Not filling the clinical profile or the number and interval time of check-up tests and consultations.
- Not valid APAP study.

**Table 1:** Number of selected patients meeting inclusion criteria. *Data are mean ± SD or n (%) values

Included patients	Breas device	Resmed device
N	30	30
Age, years	48,7 ± 10,5*	46,3 ± 11,9*
BMI	27,9 ± 7,2	28,3 ± 6,5
Sex (Male)	24 (80%)*	23(76%)*
Number of 2-nights APAP study	4	12
Number of 3-nights APAP study	26	18
IAH in HST previous to 1 st consultation	43,75	37,59

**Table 2:** Exclusion criteria (number and % of patients)

Excluded patients	54/114	47,36%
Non compliants or not enough use	22/54	40,74%
Not filling the clinical profile or protocol	20/54	37,20%
Not valid APAP study	12/54	22,22%

Every included subject underwent to a minimum of 2-nights APAP titration, with different devices, so we could analyse APAP parameters blindly for each patient and for each night’s data, to compute the differences between PF (and Pa) and the APAP pressures (PF–P95, PF–Pmed, Pa–P95, Pa–Pmed). We also examined whether using the first night alone, the second night alone, or the average of two nights affected the results. Baseline AHI (from the diagnostic HST) and CPAP compliance (hours/night) were recorded to explore correlations.

Statistical analysis was performed using SPSS v22.0. Since this is a retrospective study, we were unable to control either the size or distribution of the groups. We therefore resorted to standard statistical tests that are reliable and recognized in similar clinical research.

Specifically, we took a flexible approach: first, we checked the normality of the data using histograms and measures of skewness and kurtosis. Depending on the results, we applied parametric or nonparametric tests. Thus, Student’s t-test was used for paired samples when the assumptions of normality were met, and the Wilcoxon test was used in the opposite cases. For correlations, Pearson’s test was applied when the distribution was approximately normal, and Spearman’s test was applied when it was not.

We first tested normality of the pressure differences. Paired comparisons (paired t-test or Wilcoxon signed-rank test, as appropriate) were used to compare APAP-derived pressures (P95, Pmed) with the clinician pressures (Pa and PF). When comparing Breas device versus Resmed device, or first vs. second night, independent tests were used. Significance was set at *p* < 0.05. We also calculated Pearson or Spearman correlations between baseline AHI and compliance. Descriptive statistics are reported as mean ± SD or percentage as appropriate. No table directly comparing APAP and CPAP values was constructed, to focus on narrative analysis.

## Results

### Patient characteristics and titration protocol

**Fig. 2:**
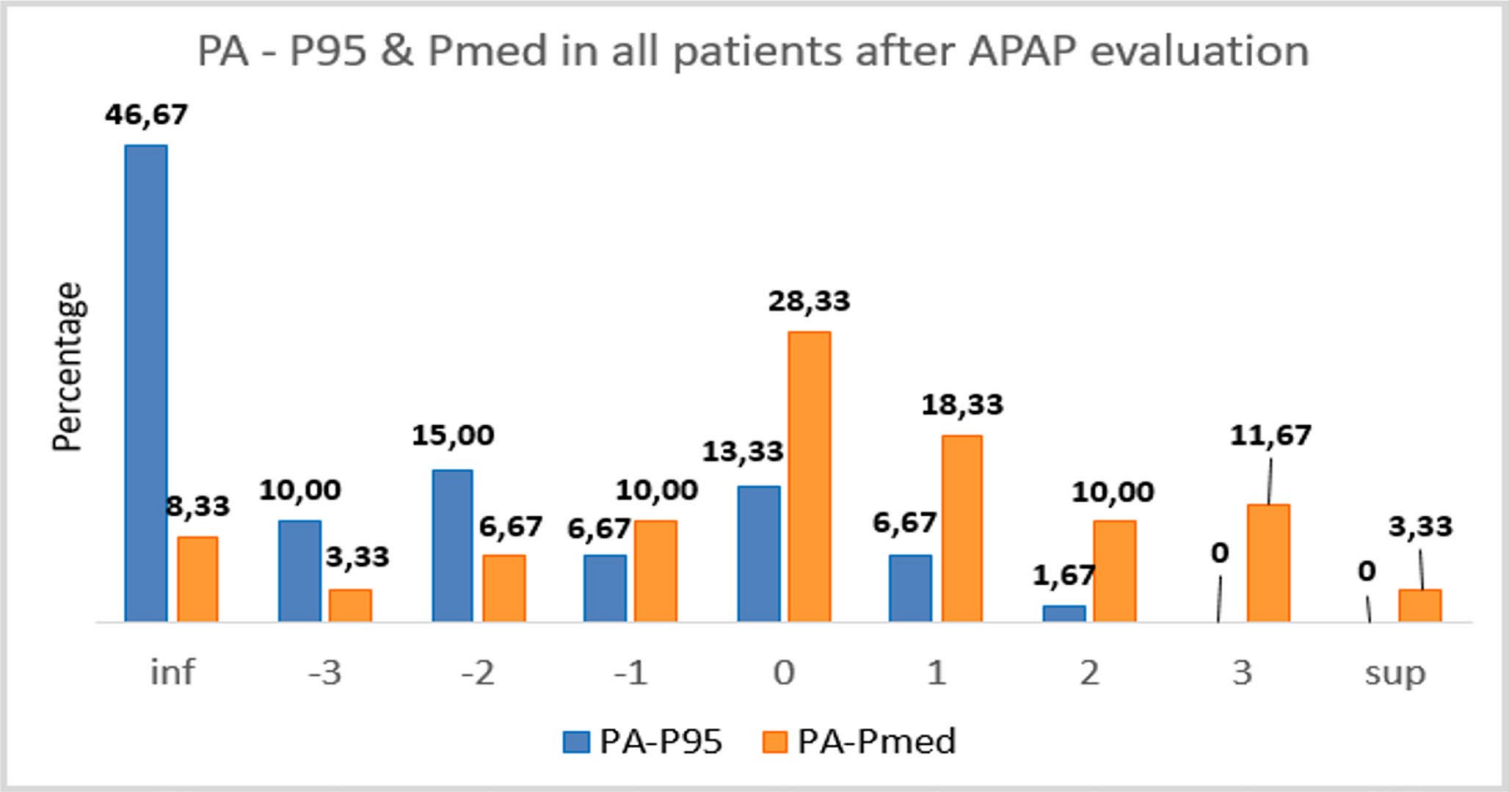
Percentage of differences between Pa and P95 or PMed grouped in intervals with a unit of length and centered on the integer values of the abscissa axis. (Ex: category 0 contains values between −0.5 and 0.5). "SUP" label indicates values greater than 3.5 and "INF" label indicates values less than −3.5

**Fig. 3:**
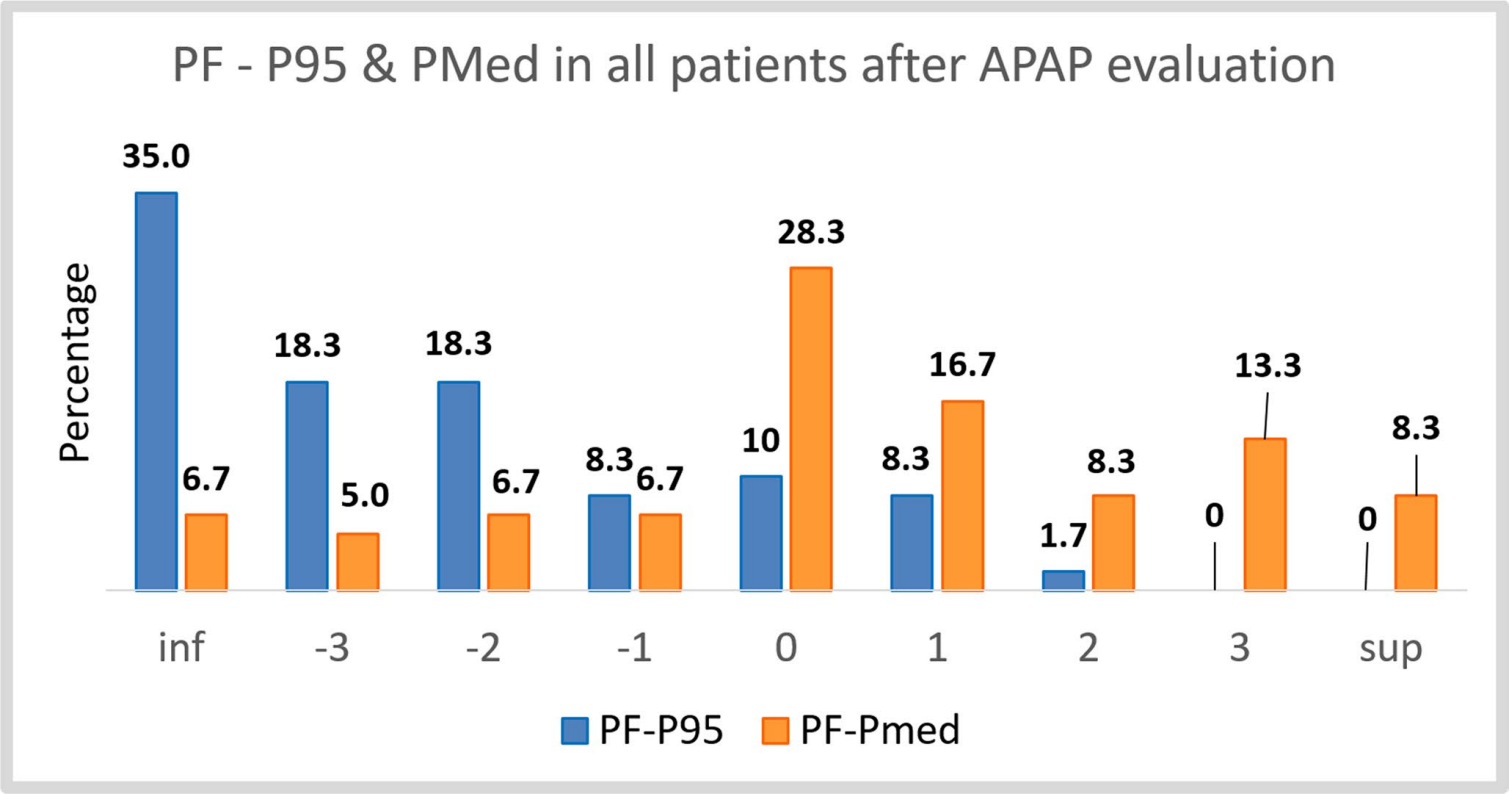
Percentage of differences between PF and P95 or PMed grouped in intervals with a unit of length and centered on the integer values of the abscissa axis. (Ex: category 0 contains values between −0.5 and 0.5). "SUP" label indicates values greater than 3.5 and "INF" label indicates values less than −3.5

The 60 included patients (30 on Breas device, 30 on Resmed device; mean age 48 ± 10 years, 80% male, mean BMI 28 ± 7) had similar baseline OSA severity (mean AHI ~ 40/h). As per protocol, Breas device studies lasted 3 nights in 26 patients and 2 nights in 4 patients, while Resmed device studies lasted 3 nights in 18 patients and 2 nights in 12 patients (Table 2). In total, 158 nights of APAP data were reviewed. (Fig. 2) The final fixed CPAP pressures (PF) determined by clinicians ranged from 8 to 16 cmH₂O (mean ~ 11 cmH₂O) (Fig. 3).

### Comparison of APAP-derived pressures to clinician pressures

**Fig. 4:**
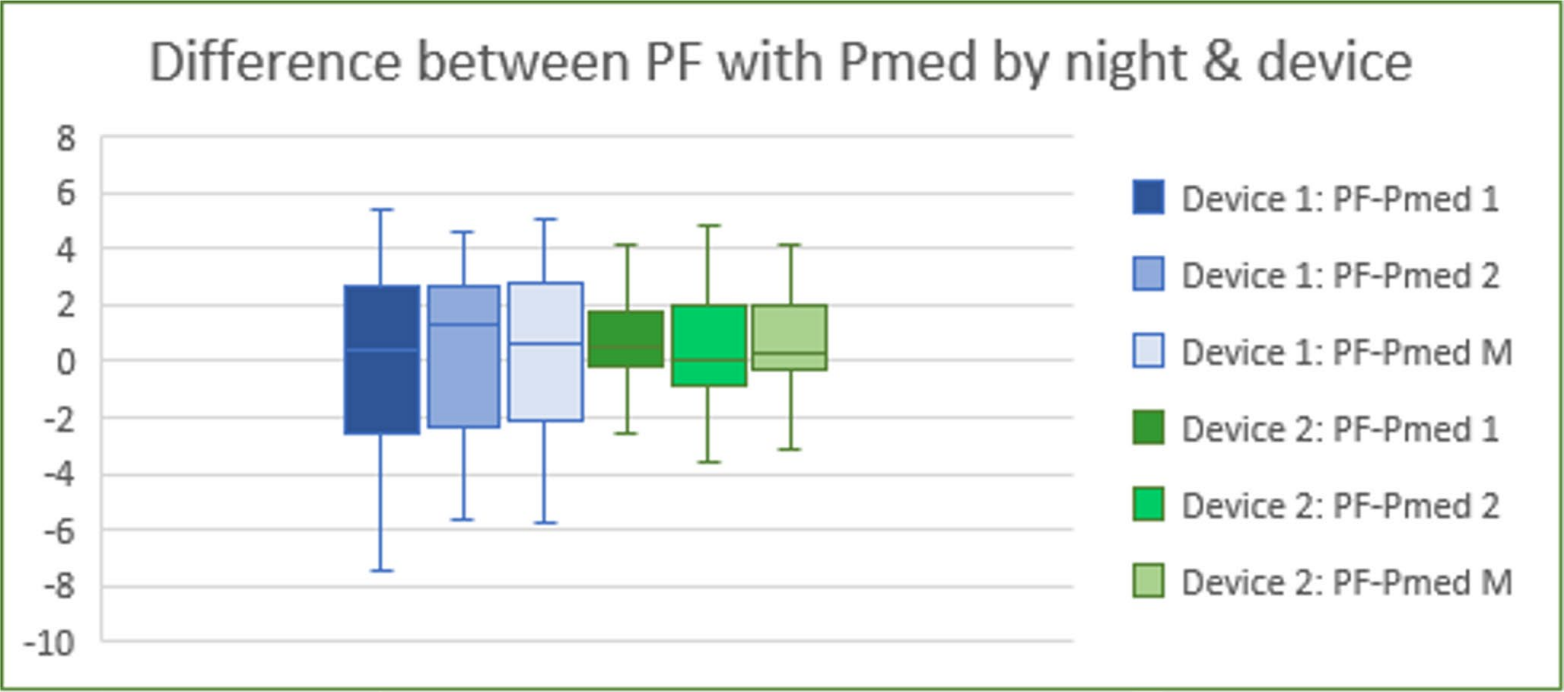
Representation of quartiles and mean of differences between PF and PMed got only on first night of APAP (PF-PMed 1), only on second night of APAP (PF-PMed 2) or considering the average of first and second nights (PF-PMed M), depending on Breas device (1) or Resmed device (2)

**Fig. 5:**
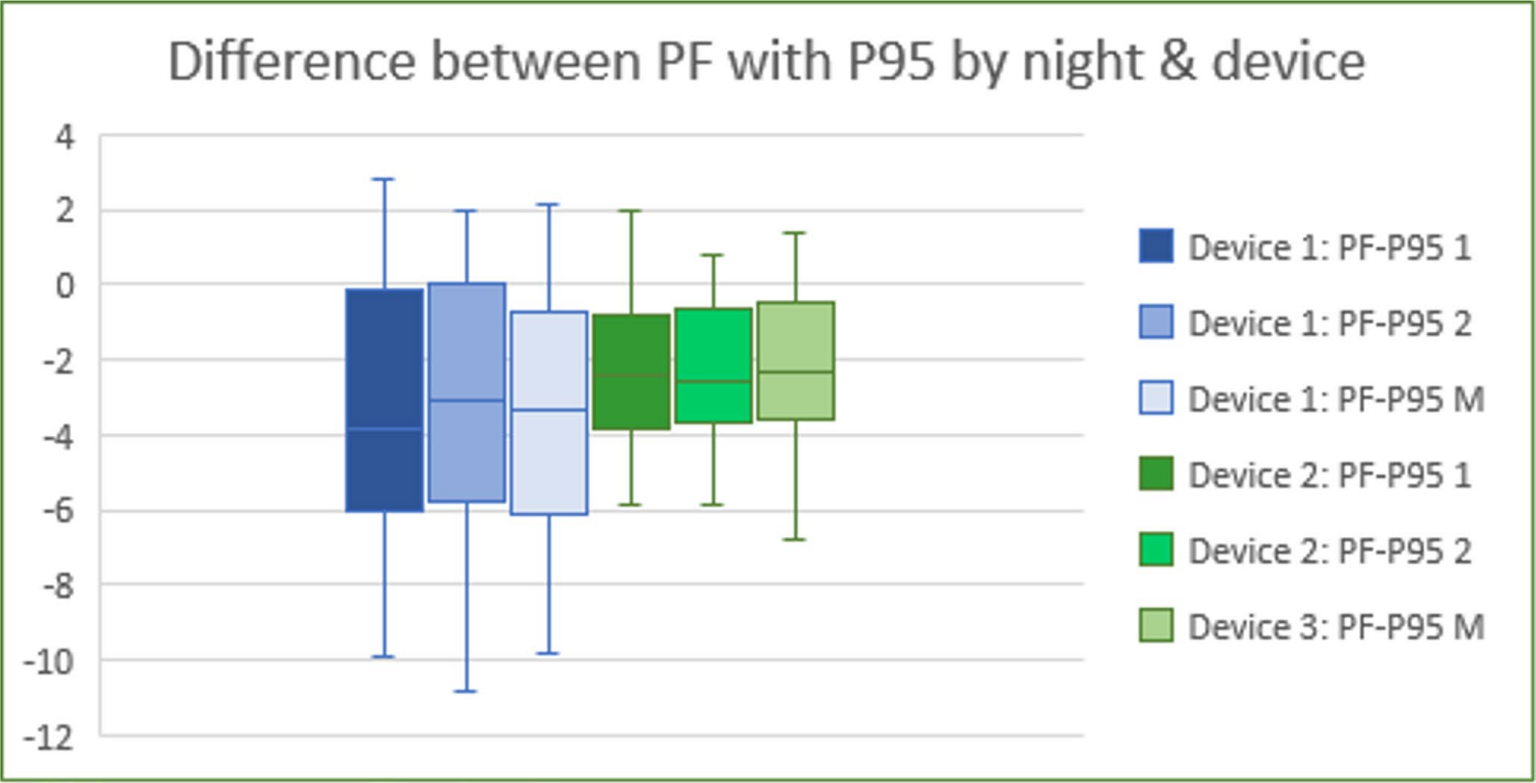
Representation of quartiles and mean of differences between PF and P95 got only on first night of APAP (PF-PMed1), only on second night of APAP (PF-PMed 2) or considering the average of first and second nights (PF-PMed M), according to APAP Breas device (1) or Resmed device (2)

**Fig. 6:**
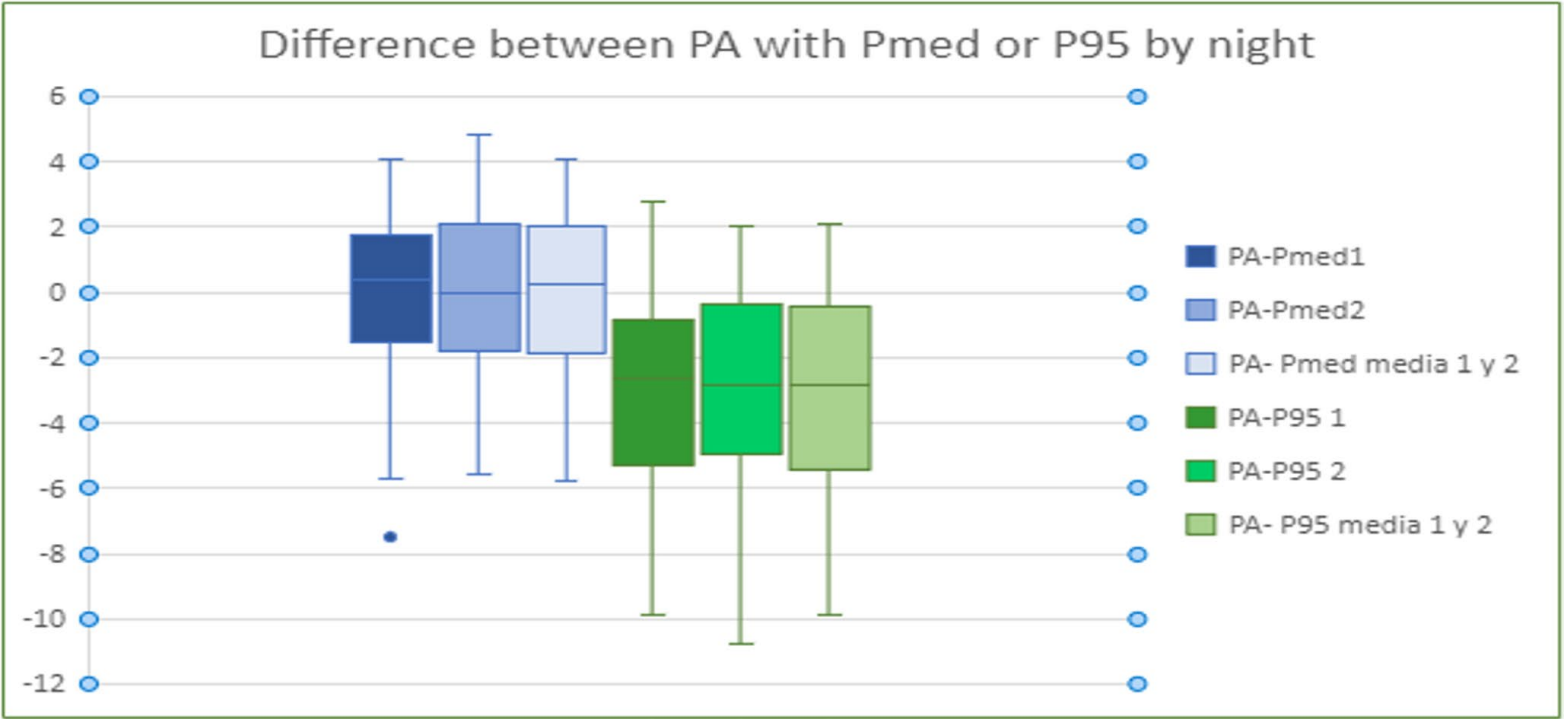
Representation of quartiles and mean (media) of differences between Pa and Pmed or P95 got only on first night of APAP (Pa-PMed1), only on second night of APAP (Pa-PMed 2) or considering the average of first and second nights (Pa-PMed M), regardless of the APAP device

We first examined how the two APAP metrics related to the pressures ultimately chosen. Figures 4 and 5 show the differences between the final pressure after follow-up (PF) and APAP pressures. PF was typically closer to the APAP median: more than half the patients had PF within ± 1 cmH₂O of Pmed (see figure 4), whereas P95 tended to overshoot PF by > 3 cmH₂O in many cases (see fig. 5). Besides, fig. 6 shows the distribution of differences between the clinician-assigned pressure after APAP review (Pa) and the APAP authomatic pressures (Pmed and P95). Notably, over 50% of patients had Pa within ± 1 cmH₂O of the APAP median pressure from at least one night. By contrast, P95 often overshot Pa in 53% of patients, P95 exceeded Pa by > 3 cmH₂O (Fig. 3). On average, the APAP median pressure was closer to Pa than P95.

When we formally tested these differences, we found that the mean PF–Pmed difference was smaller (closer to zero) than PF–P95. Paired analysis confirmed that PF differed significantly less from Pmed than from P95 (*p* < 0.05). In practical terms, using Pmed as the basis for therapy would more often match the clinician’s chosen pressure, whereas relying on P95 alone risked “supratitration” in our sample.

### Effect of device and number of nights

**Fig. 7:**
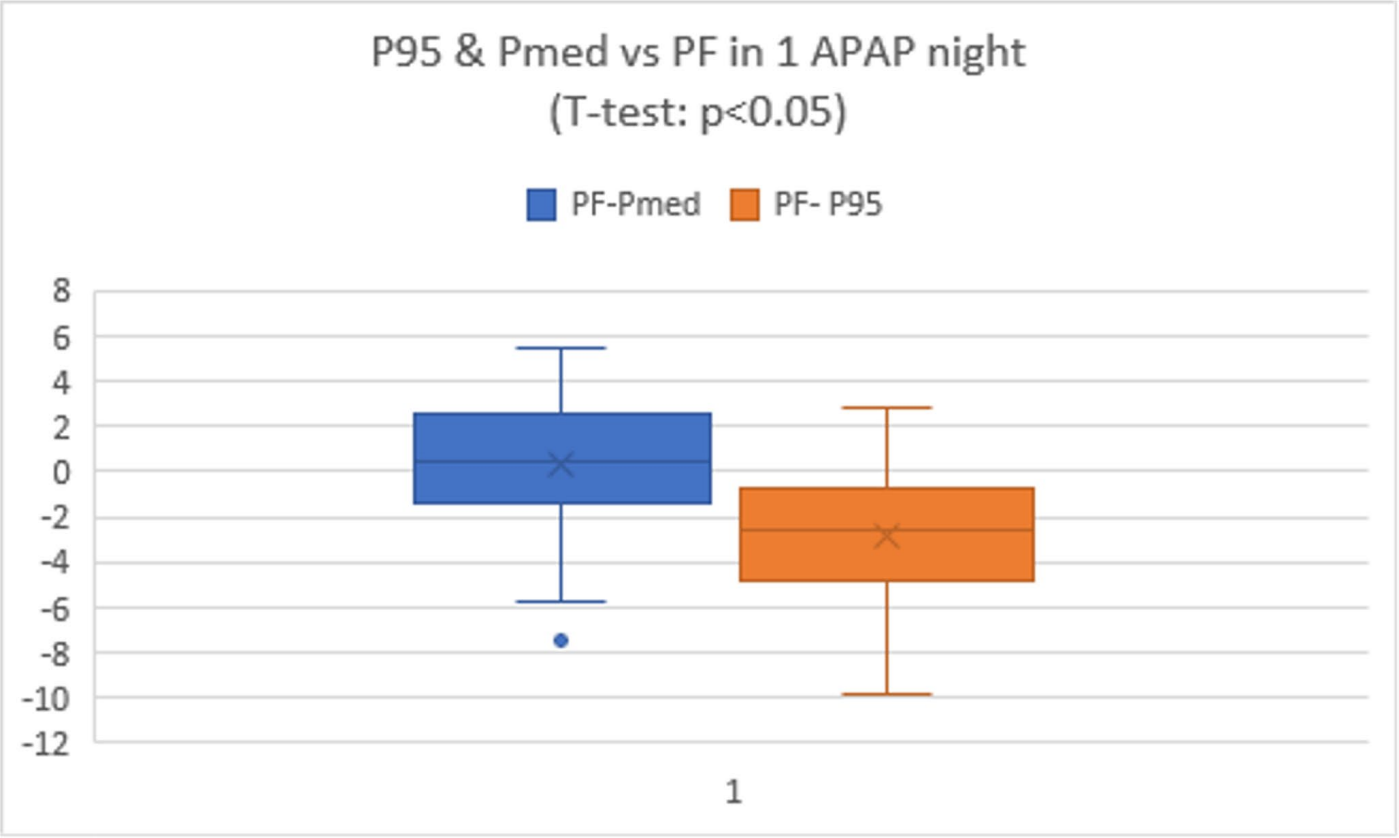
Representation of quartiles and mean of differences between PF and P95 or PMed. Applied T-test to compare the mean differences between both devices: Breas or device 1 and Resmed or device 2

**Fig. 8:**
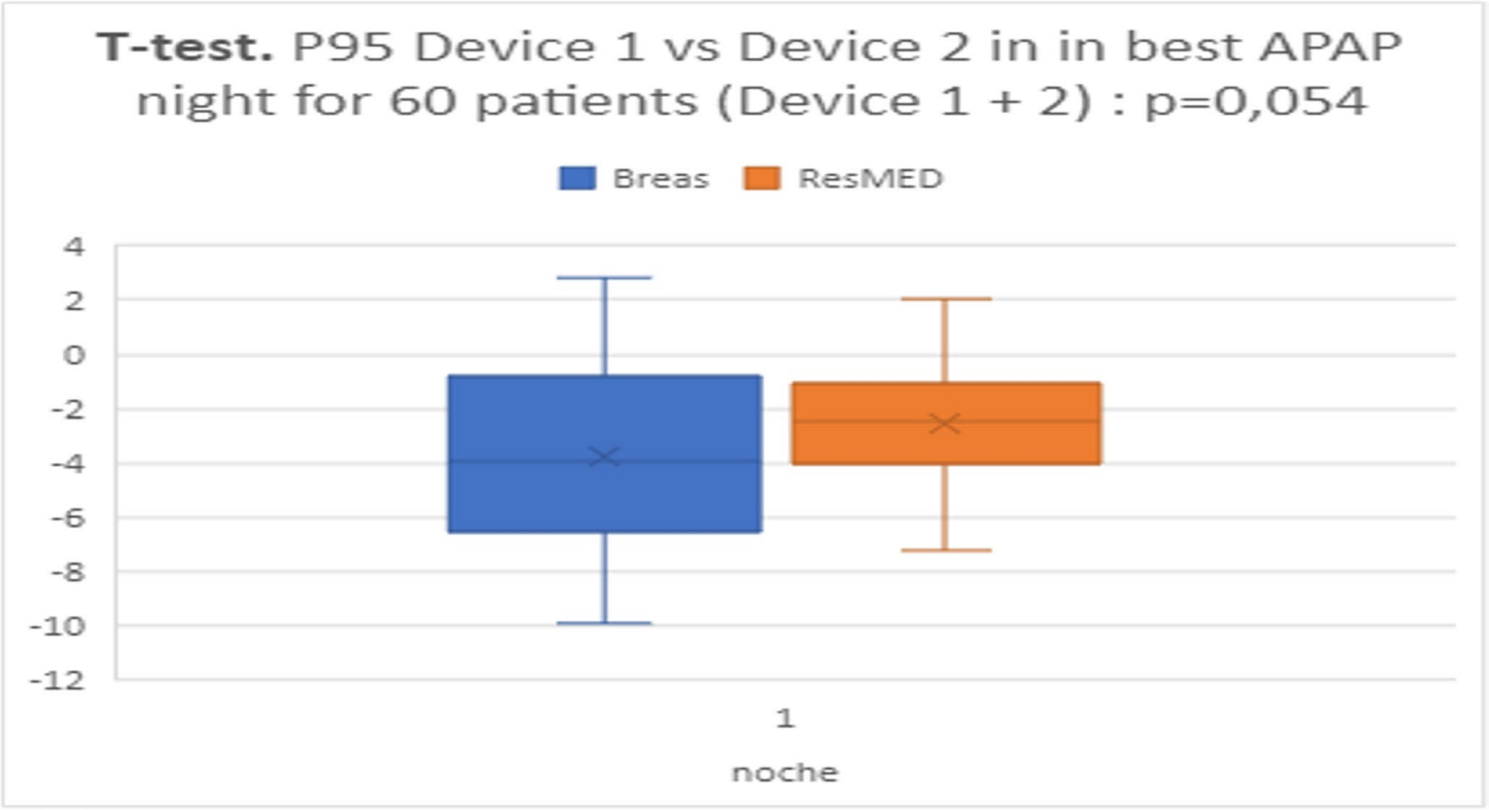
Representation of quartiles and mean of differences between Pa and P95 or PMed. Applied T-test to compare the mean differences between both devices: Breas or device 1 and Resmed or device 2

We next examined whether device type or number of nights altered these findings. When comparing Breas (device 1) vs. Resmed (device 2), there was a trend toward better agreement (smaller PF–Pmed) with Resmed device, although the sample was small. Significantly, we found that one night of APAP (particularly using Resmed device’s algorithm) provided an estimate of PF that was statistically indistinguishable from that using two nights. In other words, adding a second night did not significantly improve the prediction of PF (*p* > 0.05). Thus, in our cohort, a single-night Pmed (from Resmed device) yielded a closer match to PF than a single-night P95 (especially from Breas device). Fig. 7 and 8 illustrate boxplots of PF–Pmed and PF–P95 differences by night and device, supporting these comparisons.

### Correlation with AHI and compliance

**Fig. 9:**
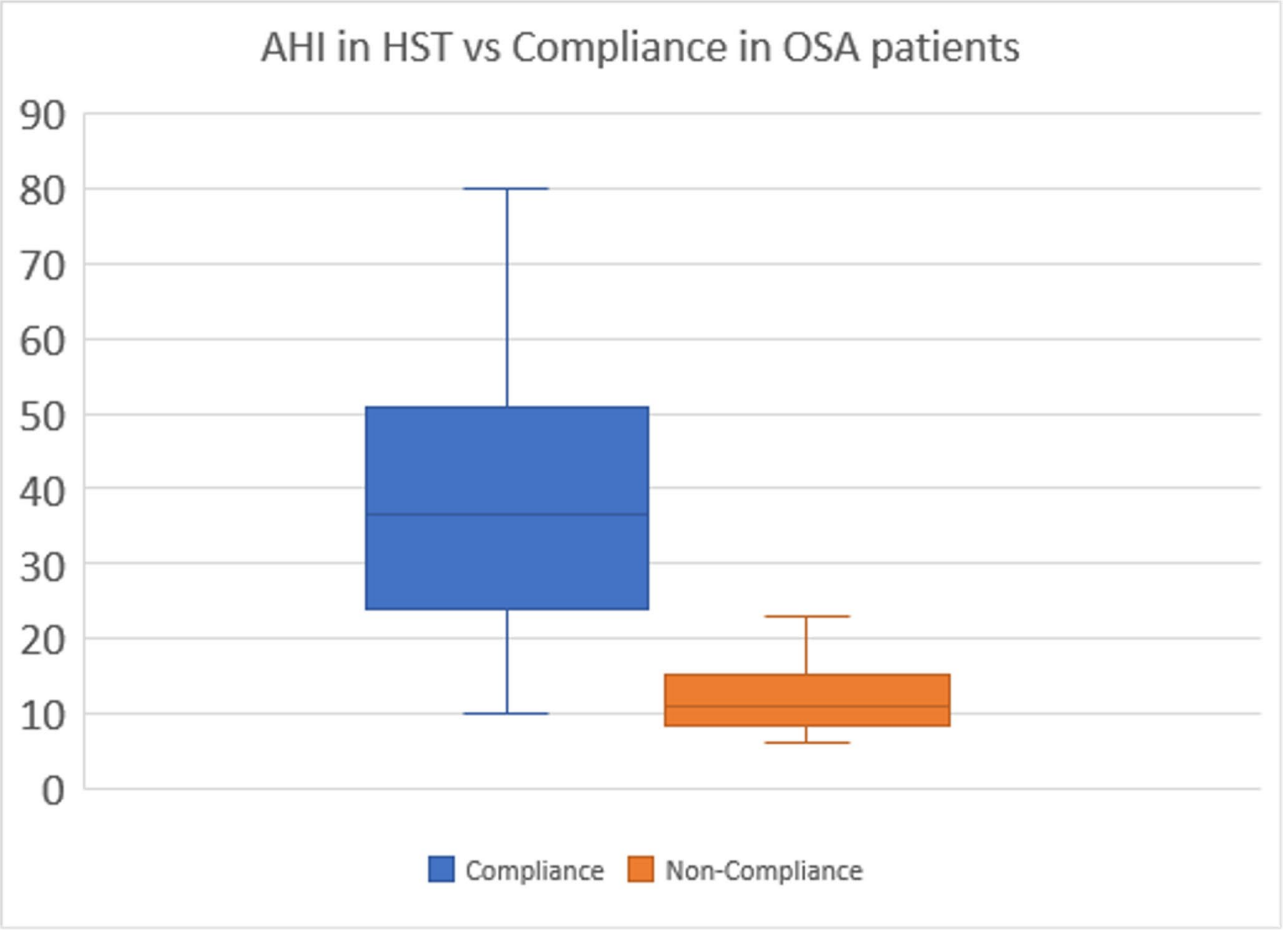
Representation of quartiles and mean of differences between PF and P95 or PMed.Applied T-test to compare the mean differences between both parameters

We also explored whether baseline OSA severity predicted CPAP adherence. AHI on the initial sleep study was positively correlated with average CPAP usage (*r* ≈ 0.30, *p* < 0.05); patients with higher AHI tended to use their therapy more hours/night. Fig. 9 shows this scatterplot (baseline AHI vs. adherence). This finding aligns with prior reports that more severe OSA often motivates better compliance. No other baseline factor (age, BMI) was significantly related to adherence in our sample.

### Statistical methods

Differences between paired pressures (e.g. PF vs. Pmed) were analyzed with paired tests as noted above. For example, we used a paired t-test to compare the mean PF and mean Pmed (or the equivalent Wilcoxon test if normality was not met). All statistical tests were two-tailed with α = 0.05. Correlations used Pearson’s or Spearman’s method depending on distribution. All analyses were pre-planned given the retrospective design; we do not present post-hoc multiple comparisons beyond those necessary to address the study questions.

## Discussion

In the present study with in a real-world Spanish cohort analysed in order to compare the CPAP pressure decission by the clinician with regard to APAP parameters, we found that the median pressure from a single night of APAP titration often approximated the clinician-determined CPAP setting, whereas the 95th-percentile pressure frequently overestimated it. Notably, using two nights of APAP did not significantly change this conclusion. These results suggest that one-night APAP titration focusing on median pressure may be sufficient in most of cases. To our knowledge, few studies have directly examined this in a pragmatic setting, especially in Spain, where home APAP titration is not as formalized or standardized as in other countries.

Our findings must be interpreted taking in account the particular context and limitations. First, APAP devices from different manufacturers implement distinct algorithms. In practice, each APAP-derived pressures can vary by device brand. In our study, Resmed device median pressures happened to align better with physician choices (final pressure) than Breas device, but this might reflect device-specific sensing or patient-device matching. Besides, clinicians should be aware of these algorithmic differences when interpreting APAP reports and the previous 2–3 months trial period when the hysteresis phenomenon in upper airway (which accounts for a decrease in the effective positive pressure level) has been reached and significant [30]. Indeed, it is often recommended that patients continue on the same brand of PAP for titration and therapy [27]to maintain consistency. In fact, the APAP pressure has been found to be 1 to 1.5 cm of H_2_O lower than the one obtained by manual titration with PSG [24].

Second, our protocol reflects a Spanish healthcare model where 2 or 3 nights of APAP (called also “auto-CPAP”) testing for Pf determination are standard. This differs from other countries (e.g. certain U.S. workflows) where a single-night or split-night study may be used, or where APAP is the treatment itself. Interestingly, we found no statistical advantage to multiple-night studies in predicting the final pressure. This aligns with the idea that night-to-night variability in pressure needs may not be large for many patients. However, some prior reports have recommended multiple nights to account for variability. Our results suggest that if resources are limited, a single well-executed night of APAP may suffice for many patients, provided the device algorithm is reliable, at least after an adaptation period to a CPAP device.

### Limitations

Several factors limit our conclusions. The sample size is relatively small (60 patients total, 30 per device group), which reduces statistical power and may limit generalizability. We did not include a direct comparison to in-lab PSG titration (the gold standard), so we cannot say how APAP would have matched a formal sleep lab pressure. Also, by design, we selected patients with stable CPAP pressures over one year; this means we excluded those whose optimal pressure was changed (perhaps because they had variable weight or airway status). As a result, our cohort may represent patients with more consistent OSA physiology. This selection improves internal consistency but limits generalizability: our findings may not apply to patients with unstable conditions or frequent pressure adjustments.

Additionally, the imbalance in “2-night vs 3-night” titrations between devices could introduce bias. Breas device patients mostly had 3-night studies, whereas Resmed device patients often had only 2 nights. While our analysis suggested the extra night did not change the outcome, the non-random allocation of nights is a potential confounder. We also lacked systematic data on mask leaks beyond excluding gross leakage; mild differences in leak could have influenced APAP readings.

Moreover, we did not provide detailed demographic breakdown due to limited variables (e.g., we did not stratify by gender or co-morbidities beyond the inclusion criteria). The majority of our sample was middle-aged, overweight men, which reflects typical OSA demographics but again may limit applicability to women or other subgroups. Finally, this was a retrospective real-world study, so we could not control for all variables (e.g. how strictly patients changed position or slept in supine). Prospective validation would be ideal.

### Comparison with other studies

Our conclusion that APAP can effectively replace lab titration for pressure setting is in line with consensus opinion [1]. Prior meta-analyses show no clinically significant difference in AHI or symptoms when using APAP titration instead of manual titration [27, 30]. The novelty here is focusing on which APAP metric best predicts the final pressure, and on showing that one night suffices for many patients. We also found, as others have, that automatic devices tend to recommend slightly lower average pressure than manual titration would [27]. Importantly, we document that median pressure may avoid the “pressure overshoot” tendency of P95. This insight is practical: clinicians often default to the 95th percentile, thinking it covers most events, but our data suggest a balanced approach using median may align better with clinical judgement.

Finally, our finding of a correlation between baseline AHI and CPAP adherence is consistent with literature indicating patients with more severe OSA (higher AHI) are more symptomatic and thus more adherent to therapy. We confirmed this relation in our cohort (see Figure F), reinforcing that initial severity can predict likelihood of good use.

## Conclusion

In summary, this study of 60 Spanish OSA patients suggests that a single night of APAP titration, using the median pressure value, can often guide an appropriate fixed CPAP prescription. We highlight that device algorithms vary and that reliance on the 95th percentile alone may lead to overestimation of needed pressure.

### Key implications

Sleep specialists in similar settings might consider emphasizing the median pressure from an APAP report when setting CPAP, and recognize that one night of good data may be sufficient. However, careful follow-up is still needed, as we recommend confirming the choice over time. Limitations (small sample, selected patients, no PSG) mean further research is warranted, ideally larger prospective studies or randomized trials comparing APAP-based titration to laboratory methods. Until then, our findings provide a practical real-world reference for sleep specialists using APAP to set CPAP pressure.

## Data Availability

The data that support the findings of this study are not openly available due to reasons of sensitivity and are available from the corresponding author upon reasonable request. Data are located in controlled access data storage at Sagunto University Hospital.

## Acronyms

APAP: Authomatic Possitive Air Pressure
CPAP: Continous Possitive Air Pressure
OSA: Obstructive Sleep Apnea
HST: Home-Sleep Testing
PSG: polysomnography
PF: Final Pressure
Pa: APAP pressure decided by clinician
Pm: APAP median pressure
P95: APAP percentile 95 pressure
